# Safety and efficacy of intravenous thrombolysis: a systematic review and meta-analysis of 93,057 minor stroke patients

**DOI:** 10.1186/s12883-024-04000-8

**Published:** 2025-01-22

**Authors:** Mostafa Hossam El Din Moawad, Talal Salem, Anas Alaaeldin, Youssef Elaraby, Peter D. Awad, Amr Ahmed Khalifa, Ahmed El Naggar, Khaled Ashraf Mohamed, Mohamed Elhalal, Mostafa Badr, Ramy Abdelnaby

**Affiliations:** 1 Faculty of Medicine, Suez Canal University, Ismailia, Egypt; 2 Alexandria Main University Hospital, Alexandria, Egypt; 3 Faculty of Medicine, Masaryk University, Brno, Czech Republic; 4 Faculty of Medicine, Cairo University, Cairo, Egypt; 5 Faculty of Medicine, New Giza University, Cairo, Egypt; 6 Department of Public Health, Theodor Bilharz Research Institute, Cairo, Egypt; 7 Neuroradiology Department, RWTH University Hospital of Aachen, Aachen, Germany; 8 Department for Epileptology, Bonn University, Bonn, Germany; 9 Department of Neurology, RWTH Aachen University, Pauwels Street 30, Aachen, 52074 Germany

**Keywords:** Acute ischemic stroke, Tissue Plasminogen Activator, Efficacy, Safety, Intracranial hemorrhage

## Abstract

**Background:**

The definition of minor ischemic stroke (MIS) is a topic of debate, however, the most accepted definition is a stroke with National Institutes of Health Stroke Scale (NIHSS) ≤ 5. Intravenous thrombolysis (IVT) is a crucial treatment option for acute ischemic stroke (AIS) including: alteplase, recombinant human tissue-type plasminogen activator (r-tPA), and the recently approved tenecteplase. However, there is a debate regarding its safety and efficacy. Therefore, our objective was to determine the safety and efficacy of IVT in treating minor stroke patients (NIHSS ≤ 5).

**Methods:**

Using the search strategy assigned which was based on three keywords: “mild” or “minor”, “stroke”, and “intravenous thrombolysis”, we searched for eligible articles on PubMed, Web of Science, Embase, and Scopus from inception till 10th January 2024. We conducted this meta-analysis using the random effect model to account for the heterogeneity among the studies. For the dichotomous variables, we calculated the odds ratio (OR) from the event and total of these variables. While for the continuous variables, we calculated the mean difference (MD) of these variables. Pooling of OR for the occurrence of events was also conducted.

**Results:**

A total of 21 articles with 93,057 patients with MIS were included. The mean age of the participants ranged from 62.3 to 79.6. Most of the included patients had comorbidities such as hypertension, diabetes, previous stroke, smoking, atrial fibrillation, and hyperlipidemia. Of these, 10,850 received IVT while 82,207 did not. The use of IVT was statistically significant associated with 90-day modified Rankin score (mRs) 0–1 when compared with control with OR of 1.67 (95%CI: 1.46, 1.91, *p* < 0.00001) and was statistically significantly associated with improvement of NIHSS on discharge with OR of 2.19 (95%CI: 1.56, 3.08, *p* < 0.00001). In terms of safety outcomes, IVT has proven a safe profile, as there was no significant difference in intracranial hemorrhage (ICH) and mortality rates between the IVT and control groups with OR of 1.75 (95CI: 0.95, 3.23, *p* = 0.07) and 0.93 (95%CI: 0.77, 1.11, *p* = 0.41), respectively.

**Conclusion:**

Although some studies have not found any benefits of IVT in MIS patients, a substantial body of literature strongly endorses IVT as an effective and safe treatment for MIS. IVT has been shown to improve the mRs and NIHSS scores at the 90-day mark without an increased risk of ICH or mortality.

**Supplementary Information:**

The online version contains supplementary material available at 10.1186/s12883-024-04000-8.

## Introduction

Acute ischemic stroke (AIS) is a severe cerebrovascular and neurological disease that can cause serious disability, morbidity, and mortality globally [1]. It is categorized into minor or mild with National Institutes of Health Stroke Scale (NIHSS) of ≤ 5, moderate from 6 to 15, and severe more than 15 [2]. The NIHSS is a 15-item assessment tool utilized to evaluate stroke severity. Initially created in 1989, it is now a commonly utilized outcome metric in stroke research. The current National Stroke Foundation guidelines endorse the NIHSS as a reliable instrument for evaluating stroke severity in emergency departments [3]. In literature, the NIHSS score and mRs are commonly employed to assess stroke severity and patient selection in studies, with varying cutoff NIHSS scores utilized by different research groups. Our research indicated that a cutoff NIHSS score of 5 or lower is the most commonly utilized in research projects [4]. The definition of minor ischemic stroke (MIS) is controversial, however the most accepted definition is a stroke with NIHSS ≤ 5 [5]. Nonetheless, a threshold NIHSS of 3 points or lower has been deemed appropriate for defining mild stroke in many studies [6], as patients within this cohort had improved outcomes after 90 days. Other studies characterize a mild stroke as an mRs score of < 2, acknowledging that the mRs provides insights into mortality risk and impairment, hence more effectively identifying patient needs post-hospital release [7]. Various research has presented several definitions for mild stroke; however, no consensus has been reached. MIS is common and is usually undervalued much by doctors or patients due to its mild symptoms. However, if the disease is not controlled at an early stage, it could rapidly deteriorate, increasing fatality and disability rates significantly [8]. Upon admission, approximately 50% of stroke patients experience a mild stroke [9, 10]. Additionally, roughly 30% of these patients have a handicapped outcome after 90 days and are unable to walk around on their own after discharge [11, 12].

Intravenous thrombolysis (IVT) is a treatment option for MIS. Alteplase, recombinant human tissue-type plasminogen activator (r-tPA), and tenecteplase, modified tPA with longer half-life, are fibrinolytic drugs used in IVT. IVT provides quick initiation of therapy, low costs, and requires no special equipment for treatment. Hence, aids in controlling AIS at an early stage. According to a study, alteplase treatment significantly improved AIS cases [13].

Four out of ten patients who got IVT therapy in national US practice had modest impairments, according to a previous study [11], which indicated that more than half of AIS hospitalizations had these conditions. Ninety days after a minor stroke, more than one-third of patients who did not receive alteplase treatment experienced functional impairment, despite only experiencing modest neurological symptoms at first [6, 14, 15]. As a result, alteplase was suggested as a treatment for AIS individuals who had minor disabilities in the 2019 guidelines [16]. In order to lessen the possibility of post-stroke impairment, alteplase treatment for mild strokes has grown recently [17, 18].

Previous studies have demonstrated that IVT is associated with short-term outcomes, such as home release, independent ambulation, or discharge with a good functional outcome measured by modified Rankin score (mRs) [11, 19, 20]. The mRs has served as the gold standard for assessing stroke outcomes in clinical studies for many years. This ordinal scale, which assesses patient impairment from 0 (no symptoms) to 6 (death), effectively encompasses the entire range of functional states while maintaining intuitive simplicity [21]. In the examination of somewhat higher baseline NIHSS, several observational studies indicated that IVT was related with good 90-day outcomes [10, 12, 22]. But in the Potential of rt-PA for Ischemic Strokes with Mild Symptoms (PRISMS) trial, alteplase was found to be equally safe and effective as aspirin for a favorable functional result after 90 days in non-disabling MIS (NIHSS ≤ 5). Rather, the probability of symptomatic intracerebral hemorrhage (sICH) was increased threefold by alteplase [13]. However, this trial ended prematurely and yielded little substantial evidence. Therefore, the subject of whether or not to use IVT to treat patients who have had mild or minor strokes has been debated in the modern era. Consequently, our goal was to determine whether IVT is safe and effective for treating minor stroke patients (NIHSS ≤ 5).

## Methods

We conducted this study according to the principles of systematic reviews and meta-analysis indicated by the Preferred Reporting Items for Systematic Reviews and Meta-Analyses (PRISMA) guidelines [23] and the Cochrane Handbook for Systematic Reviews of Interventions [24].

### Database searching

Using the search strategy assigned which was based on three keywords: “mild” or “minor”, “stroke”, and “intravenous thrombolysis”, we searched for eligible articles on PubMed, Web of Science, Embase, and Scopus from inception till 10th January 2024. (Supplementary Table 1) The resulting articles were transferred to EndNote for the removal of duplicates before starting the screening process.

### Inclusion and exclusion criteria

We included the articles that fulfilled the inclusion criteria as follows: randomized controlled trials (RCTs) or observational studies (cohort, and case–control) investigating the safety and/or efficacy of the use of IVT in patients with minor or mild stroke (NIHSS ≤ 5) and comparing it with control patients who didn’t receive IVT. We didn’t exclude patients with comorbidities as stroke is mostly caused by different cardiovascular diseases so most of the included patients had comorbidities such as hypertension, diabetes, atrial fibrillation, smoking, and hyperlipidemia. We excluded any groups with higher NIHSS, case reports, case series, and reviews. We also excluded studies who didn’t investigate the safety or efficacy outcomes.

### Screening

After removal of duplicates, the screening process was done blindly and independently by four authors and any disagreement was solved by a senior author. The first step was title and abstract screening to indicate the eligibility of articles, then the eligible articles were assessed again by full text to make sure that they fit into the study according to the desired outcomes, and the inclusion criteria. After screening, the conflicts were assessed and the authors resolved them by consensus but if the conflict persists, a senior author resolved it.

### Data extraction

Using Microsoft Excel sheets, four authors working in a blind manner extracted the data in the prepared data extraction sheet. Baseline data included study design, country, age, sample size, gender, stroke history, and comorbidities such as hypertension, diabetes, atrial fibrillation, smoking, and hyperlipidemia. The outcome sheet included the total and event for improvement in mRs, and NIHSS in addition to odds ratio (OR) of improvement. We also extracted the mean, and standard deviation (SD) of NIHSS before and after treatment with IVT and in the control group.

### Quality assessment

Quality assessment for observational studies was conducted using the New Castle Ottawa Scale (NOS) which assigns every study a maximum of 9 stars and a minimum of 0 stars. Each question can be given a star or not except the comparability one which can be given zero, one, or two stars. If the study gets 0–3 stars, it is considered of having low quality, 4–6 stars of moderate quality, and 7–9 stars of high quality [25]. We employed the Cochrane risk-of-bias instrument (RoB 2.0) [26], which has five domains with corresponding sets of questions: randomization, variations from intended interventions, missing outcome data, outcome measurement, and selection of the reported result. These inquiries have the following responses: "yes," "no," "possibly yes," "possibly no," and "no information." After that, a graphic is used to aggregate the data and identify one of three bias levels: low risk, some concerns, or high risk. Only when each of the five domains is rated as low risk separately can a study qualify as having an overall low risk of bias. The study is deemed to have some concerns if any domain expresses some concerns. However, the study is classified as having a high risk of bias if any domain is deemed to be at high risk or if several domains raise some concerns.

### Statistical analysis

Using Review Manager version 5.4 software [27], we conducted this meta-analysis using the random effect model to account for the heterogeneity among the studies. For the dichotomous variables, we calculated the OR from the event and total of these variables. While for the continuous variables, we calculated the mean difference (MD) of these variables. We also compared the change of NIHSS in drug vs control using MD. Pooling of OR for the occurrence of events was also conducted. An OR greater than one is interpreted as increased odds or risk of this outcome, while less than one means decreased odds of occurrence or lower risk. We applied a confidence interval (CI) of 95% and the results were considered significant if the *p*-value was less than or equal to 0.05. Therefore, a *p*-value of 0.05 or less is associated with statistically significant results and important clinical implications. The heterogeneity was assessed using I^2^ and *p*-value of 0.05 as well.

### Sensitivity analysis

The random-effects model was chosen because it accounts for variability between studies, assuming that the true effect sizes are not identical but follow a distribution. This model allows for a more generalized inference across diverse study settings. The studies differ from each other regarding different patients’ characteristics such as demographics, drugs used, comorbidities, severity of stroke, and others. To better understand the heterogeneity causes, we conducted sensitivity analysis using the leave-one-out method on OpenMetaAnalyst [28] to investigate which studies caused heterogeneity present in the heterogeneous outcomes.

## Results

### Database searching and screening

**Fig. 1:**
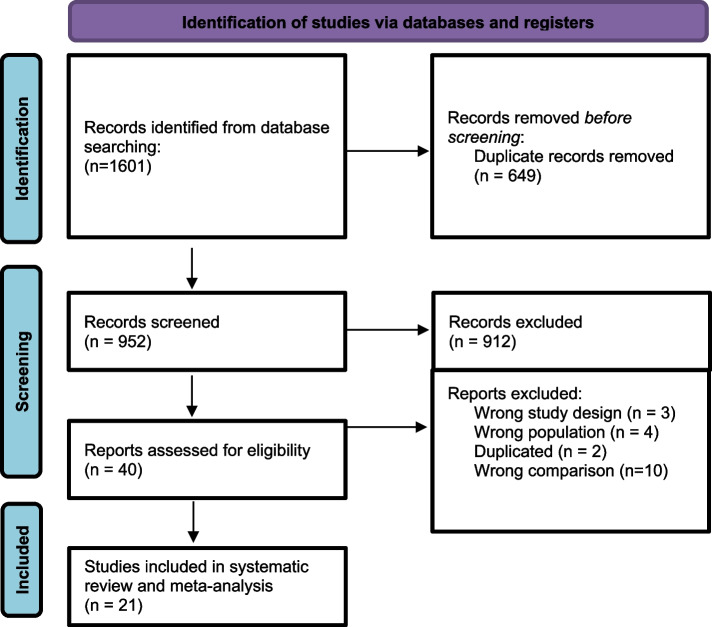
PRISMA flow diagram of database searching and screening

By applying our search strategy, the databases search process resulted in a total of 1601 including a total of 649 duplicates. After conducting the title and abstract screening for the remaining 952 articles, we included a total of 40 studies for the full text screening. A final of 21 articles [8, 10, 20, 22, 29–45] were eligible to be included in the meta-analysis (Fig. 1).

### Quality assessment

The quality assessment using NOS for the included cohort studies showed that among them, six were of high quality and 14 were of moderate quality. (Supplementary Table 2) Regarding the included RCT, risk of bias assessment using RoB 2.0 showed that it had low risk of bias. (Supplementary Fig. 1).

### Baseline characteristics

Among the 21 included studies in our meta-analysis, 20 were cohort studies while only one was an RCT. The total sample size was 93,057 with MIS, 10,850 of them taking IVT, and 82,207 patients not taking IVT. These studies were conducted in different countries including China, Germany, United States, and Australia. The mean age of the participants ranged from 62.3 to 79.6. Most of the included patients had comorbidities such as hypertension, diabetes, previous stroke, smoking, atrial fibrillation, and hyperlipidemia (Table 1).

**Table 1:** Baseline characteristics of the included articles

Study ID	Study Design	Country	Age, mean ± standard deviation		Sample size		Gender (M/F)		Hypertension, n/(%)		Diabetes, n(%)		Previous Stroke, n(%)		Atrial Fibrillation, n(%)			Smoking, n(%)		Hyperlipidemia, n(%)		
			IVT	control	IVT	control	IVT	control	IVT	control	IVT	control	IVT	control	IVT	control		IVT	control	IVT	control	
Han 2021 [37]	Cohort	China	65.19 ± 8.67	67.51 ± 11.17	96	84	56/40	57/27	73(76.04%)	64(76.19%)	22(22.92%)	39(46.43%)	10(10.42%)	19(22.62%)	11(11.46%)	4(4.76%)		41(42.71%)	40(47.62%)	__	__	
Hsia 2021 [38]	Cohort	USA	67.13 ± 15.33	73.18 ± 18.15	119	90	71/48	46/44	82	__	23(69%)	__	19(16%)	__	13(11%)	__		22(18%)	__	50(42%)	__	
Huisa 2012 [39]	Cohort	USA	66.5 ± 16.4	70.1 ± 14.5	59	74	36/23	43/31	35(59.3%)	49(66.2%)	11(18.6%)	18(24.3%)	__	__	12(19.6%)	17(23.3%)		15(25.4%)	28(37.8%)	__	__	
Li 2019 [8]	Cohort	China	79.6 ± 2.2	79.3 ± 2.1	60	60	41 /19	35 /25	48 (80.00%)	45(75%)	21(35%)	16 (26.67%)	__		__			42 (70%)	39 (65%)	18(30%)	15(25%)	
Duan 2023 [36]	Cohort	China	62 ± 2.42	62.67 ± 2.36	527	1378	361/166	971/407	342(64.9%)	841(61.0%)	110(20.9%)	322(23.4%)	90(17.1%)	310(22.5%)	40(7.6%)	114(8.3%)		172(32.6%)	428(31.1%)	39(7.4%)	116(8.4%)	
Villringer 2014 [41]	Cohort	Germany	70.3 ± 10.6	70 ± 12	62	102	36/26	64/38	__		__		__		__			__		__		
Zhong 2021 [34]	Cohort	China	68 ± 12	70 ± 14	240	221	161/79	148/73	167 (67.6%)	148 (67.0%)	52(21.7%)	60(27.1%)	41(17.1%)	48(21.7%)	51(21.3%)	35(15.8%)		94(39.2%)	61(31.0%)	__	__	
Broderick 2005 [35]	Randomized controlled trial	USA	Definition A: 67 ± 12 Definition B: 64 ± 11 Definition C: 67 ± 12 Definition D: 67 ± 11 Definition E: 67 ± 11	Definition A: 64 ± 15 Definition B: 4 ± 13 Definition C: 66 ± 12 Definition D: 64 ± 13 Definition E: 65 ± 13	Definition A: 21 Definition B: 51 Definition C: 220 Definition D: 97 Definition E: 99	Definition A: 7 Definition B: 30 Definition C: 219 Definition D: 76 Definition E: 78	__	__	__	__	Definition A: 3(14%) Definition B: 13(25%) Definition C: 44(20%) Definition D: 21(22%) Definition E: 21(21%)	Definition A: 3(43%) Definition B: 5(17%) Definition C: 49(22%) Definition D: 17(22%) Definition E: 17(22%)	__	__	__	__		__	__	__	__	
Sykora 2022 [10]	Cohort	Australia																				
		NIHSS 0–1:	70 ± 15.5	66.7 ± 20.7	703	34,402	416/287	18,975/15427	491(71%)	25,701(74.8%)	135 (19.5%)	7100 (20.7%)	98 (14.2%)	6355 (18.5%)	93(13.4%)	5207 (15.2%)		145(21%)	6141(17.9%)	384(55.5%)	19,569(57%)	
		NIHSS 2–5:	71 ± 14	71.5 ± 15	6316	39,176	3681/2635	22,204/16972	4814(77.4%)	31,875(81.5%)	1287 (20.7%)	10,735 (27.5%)	1079 (17.3%)	9528 (24.4%)	980(15.8%)	8170 (20.9%)		1167(18.8%)	7652(19.6%)	3646(58.6%)	22,864(58.5%)	
Pitaksuteepong 2020 [42]	Cohort	Thailand	64.91 ± 17.15	60.61 ± 15.21	56	138	25/31	58/80	33(58.9%)	56(40.6%)	14(25%)	30(21.7%)	10(17.9%)	18(13%)	3(5.4%)	10(7.2%)		20(35.7%)	70(50.7%)	18(32.1%)	27(19.6%)	
Sharma 2014 [45]	Cohort	USA	__	__	20	166	__	__	__	__	__	__	__	__	__	__		__	__	__	__	
Yaghi 2012 [44]	Cohort	USA	65.6 ± 12.3	66.6 ± 12.0	30	30	17/13	15/15	26(86.7%)	25(83.3%)	11(36.7%)	4(13.3%)	__	__	3(10%)	3(10%)		__`	__	14(46.7%)	13(43.3%)	
Mengel 2022 [43]	Cohort	Germany	75 ± 13 years (for total patients in the study)		201	66	151/116 (for total number of patients in the study)		__		__		__		__			__		__		
Logallo 2014 [20]	Cohort	Norway	67.3 ± 13.9	69.9 ± 14.1	158	1633	105/53	985/648	80 (51.0%)	867 (53.3%)	18 (11.6%)	221 (13.7%)	22 (14.1%)	264 (16.4%)	17 (10.8%)		229 (14.1%)	__	__	43 (27.2%)		408 (25.0%)
Urra 2013 [29]	Cohort	Spain	68.8 ± 13.8	69.0 ± 13.2	119	84	82/37	52/32	81(68.1%)	57 (67.5%)	29 (24.4%)	31 (37.3%)	__	__	18 (15.3%)		20 (23.8%)	__	__	46 (38.8%)		40 (48.2%)
Chen 2017 [30]	Cohort	China	62.3 ± 10.5	63.2 ± 10.6	134	249	86/48	159/90	92(68.7%)	156(62.7%)	24(17.9%)	59(23.7%)	23(17.2%)	89(35.7%)	10 (7.5%)		21 (8.4%)	63 (47.0%)	71 (28.5%)	__		__
Luo 2024 [31]	Cohort	China	63.7 ± 22.4	64.3 ± 11.4	619	2170	415/204	1391/779	354 (57.2%)	1328 (61.7%)	124 (20%)	447 (20.6%)	156 (25.2%)	610 (28.1%)	20 (3.2%)		48 (2.2%)	197 (31.8%)	588 (27.1%)	16 (2.6%)		26 (1.2%)
Ng 2016 [32]	Cohort	Australia	72.2 ± 14.5		34	39	23/11	22/17	21 (61.8%)	24(61.1%)	3(8.8%)	7(16.7%)	10(29.4%)	7(16.7%)	11(32.4%)		16(41.7%)	6(17.8%)	9(22.2%)	14 (41.2%)		18(47.2%)
Greisenegger 2014 [33]	Cohort	Austria	69 ± 12.6	70 ± 11.86	445	445	259/186	259/186	360(80.9%)	360(80.9%)	60(13.5%)	60(13.5%)	68(15.3%)	83(18.7%)	90(20.2%)		91(20.4%)	-	-	278(62.5%)		278(62.5%)
Choi 2015 [22]	Cohort	USA	63 ± 13	64 ± 13	194	1190	126/68	752/438	117 (60.3%)	794(66.7%)	41 (21.1%)	318(26.7%)	20(10.3%)	174(14.6%)	32 (16.5%)		205(17.2%)	81(41.8%)	508(42.7%)	58 (29.9%)		406(34%)
Laurencin 2015 [40]	Cohort	France	68 ± 15.7		170		112/58		-	-	-	-	-	-	-		-	-	-	-		-

### Meta-analysis

**Fig. 2:**
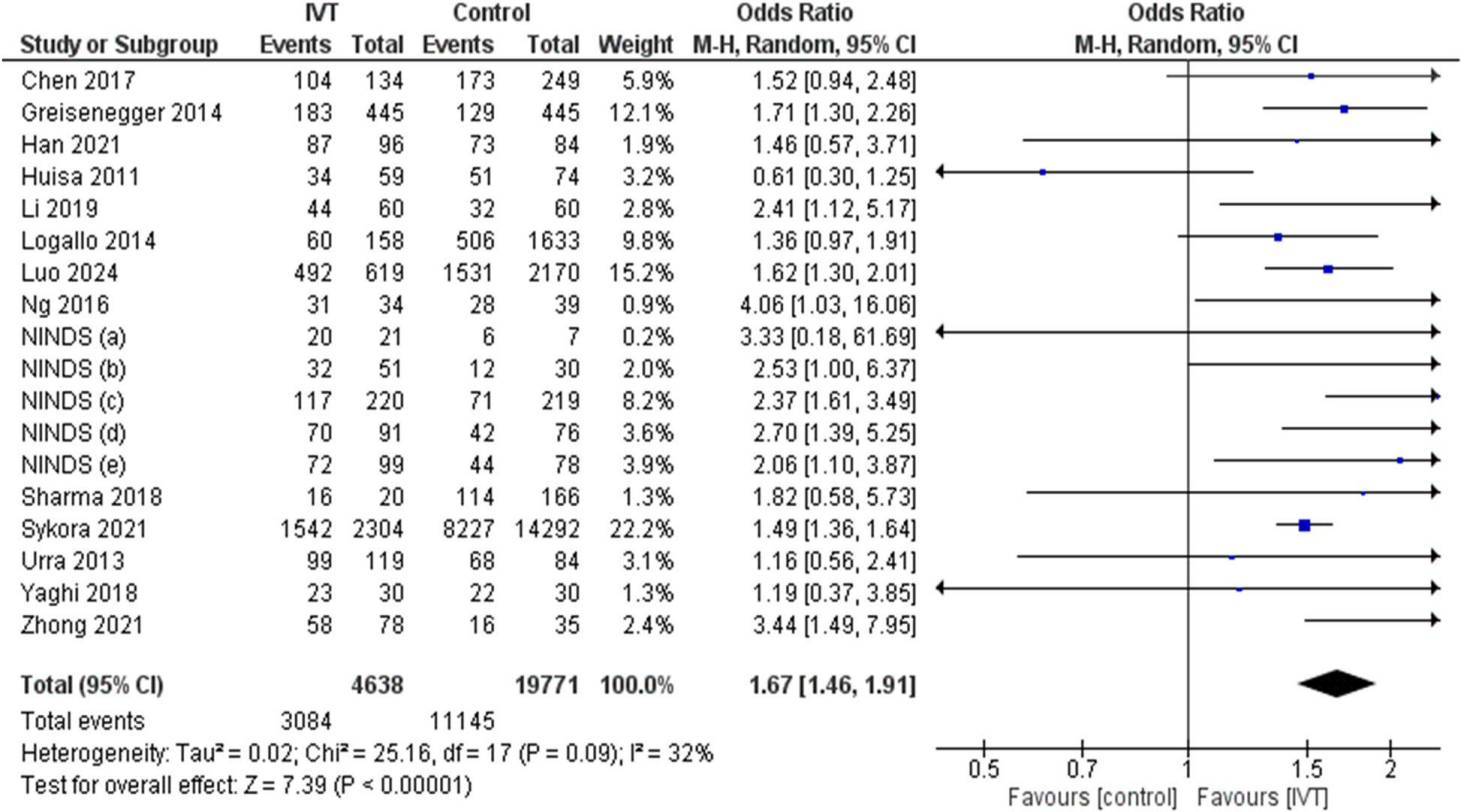
Comparison between intravenous thrombolysis and control groups regarding 90-day mRs 0–1 outcome using odds ratio. The diamond shape represents the overall forest plot, the blue region is the OR of each study and the two lines merging from it represent the confidence intervals

The use of IVT was statistically significant associated with 90-day mRs 0–1 when compared with control with OR of 1.67 (95%CI: 1.46, 1.91, *p* < 0.00001) and I^2^ = 32%, *p* = 0.09. Therefore, the use of IVT is associated with better functional outcomes at 90-day measurement compared with the control group (Fig. 2).

When using pooled OR, it was found that IVT is a statistically significant predictor of 90-day mRs 0–1 with OR of 1.62 (95%CI: 1.33, 1.98, *p* < 0.00001) and I^2^ = 60%, *p* = 0.01. (Supplementary Fig. 2).

**Fig. 3:**
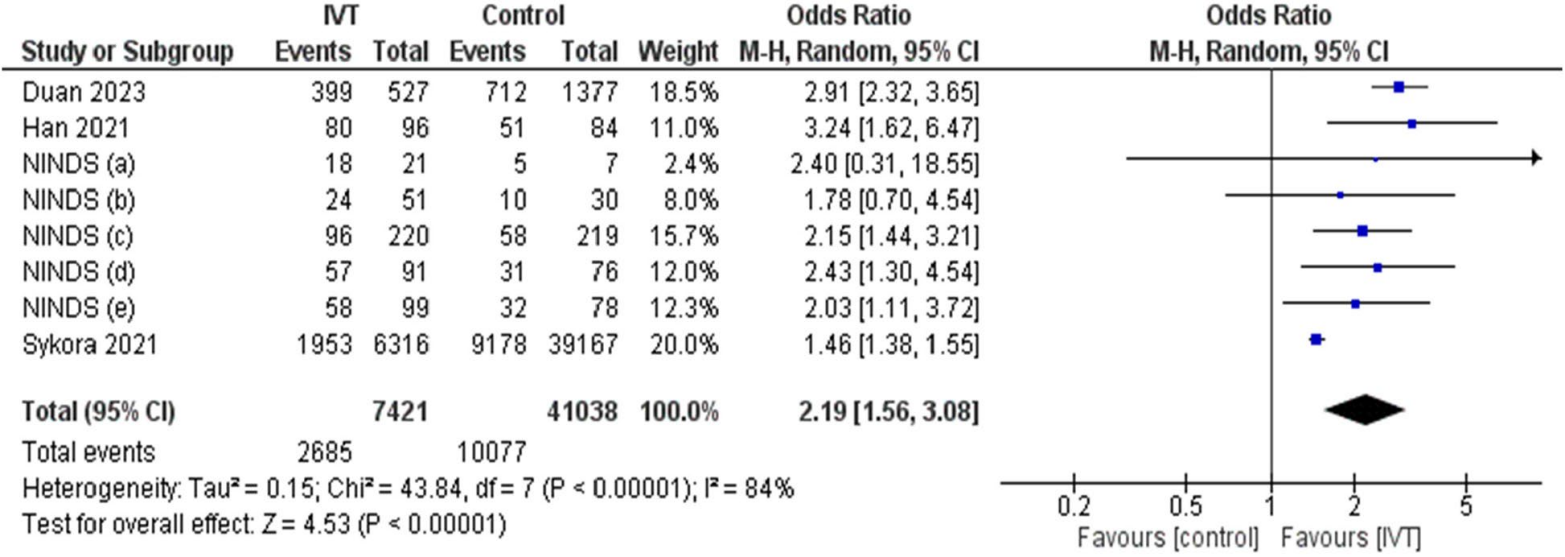
Comparison between intravenous thrombolysis and control groups regarding NIHSS improvement outcome using odds ratio. The diamond shape represents the overall forest plot, the blue region is the OR of each study and the two lines merging from it represent the confidence intervals

Also, the use of IVT was statistically significantly associated with improvement of NIHSS on discharge with OR of 2.19 (95%CI: 1.56, 3.08, *p* < 0.00001) and I^2^ = 81%, *p* < 0.00001. Thus, IVT is significantly associated with decreased stroke severity (Fig. 3).

After using the pooled analysis of OR, IVT was observed to be a significant predictor of NIHSS improvement with OR of 2 (95%CI: 1.27, 3.14, *p* = 0.003) and I^2^ = 85%, *p* = 0.001. (Supplementary Fig. 3).

**Fig. 4:**
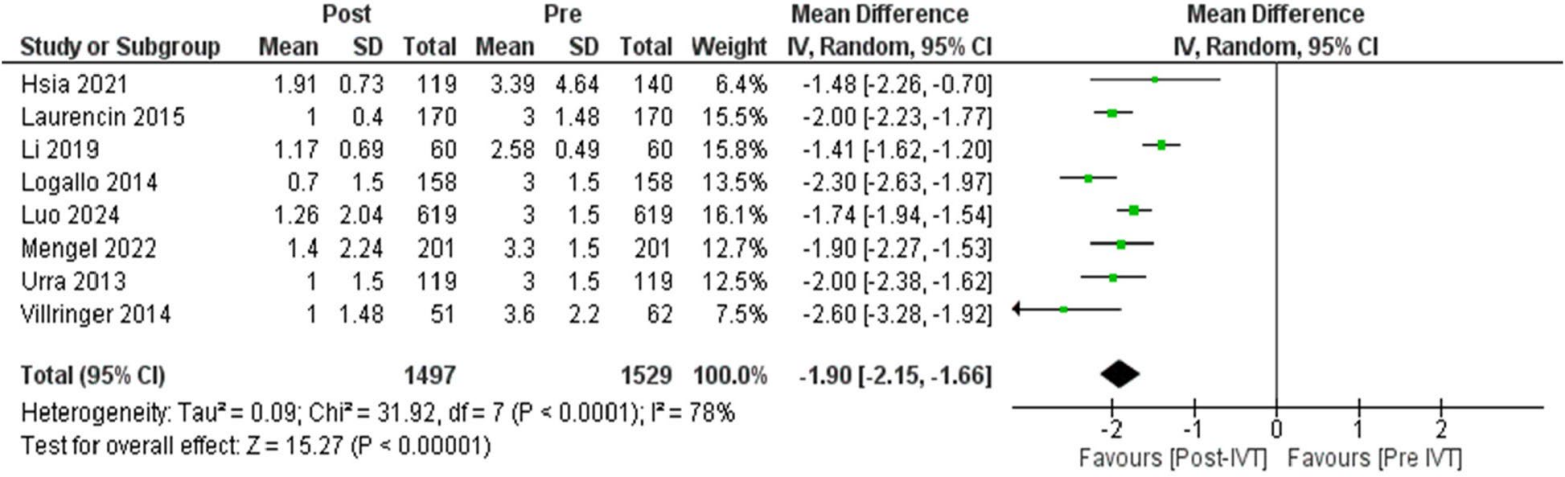
The effect of using intravenous thrombolysis on the decrease of NIHSS. The diamond shape represents the overall forest plot, the blue region is the MD of each study and the two lines merging from it represent the confidence intervals

IVT was found to decrease NIHSS on discharge compared with the values obtained before treatment with a MD of −1.9 (95%CI: −2.15, −1.66, *p* < 0.00001) and I^2^ = 78%, *p* < 0.0001. (Fig. 4) When comparing the change in NIHSS between before and after treatment, IVT was found to decrease the NIHSS more than the control group with a MD of −0.93 (95CI: −1.62, −0.24, *p* = 0.008) and I^2^ = 94%, *p* < 0.00001. (Supplementary Fig. 4).

**Fig. 5:**
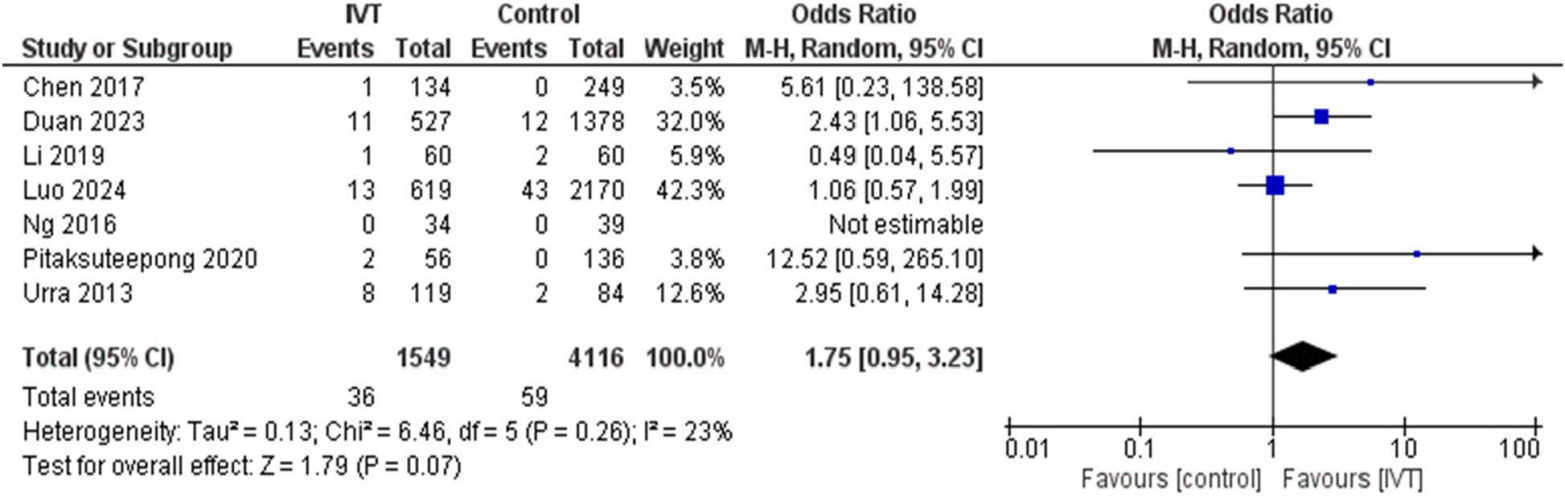
Comparison between intravenous thrombolysis and control groups regarding intracranial hemorrhage. The diamond shape represents the overall forest plot, the blue region is the OR of each study and the two lines merging from it represent the confidence intervals

**Fig. 6:**
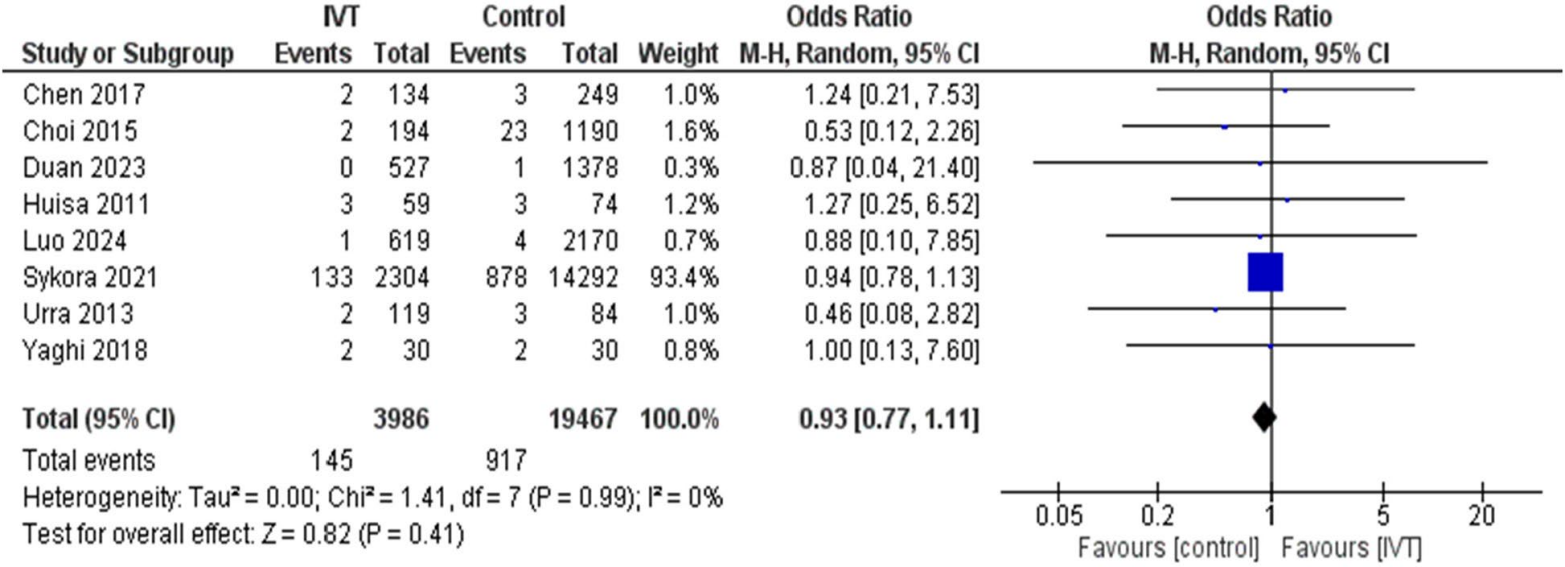
Comparison between intravenous thrombolysis and control groups regarding mortality rate. The diamond shape represents the overall forest plot, the blue region is the OR of each study and the two lines merging from it represent the confidence intervals

Regarding the safety outcomes, IVT has proven a safe profile as there was no significant difference between ICH and mortality rates between IVT and control groups with OR of 1.75 (95CI: 0.95, 3.23, *p* = 0.07) and I^2^ = 23%, *p* = 0.26, and 0.93 (95%CI: 0.77, 1.11, *p* = 0.41) and I^2^ = 0%, *p* = 0.99, respectively. Therefore, there was a comparable safety profile whether on using IVT or not. (Figs. 5 and 6).

### Sensitivity analysis

By conducting leave-one-out analysis for the 90-day mRs 0–1, it was observed that Sykora et al. [10] was the major cause of heterogeneity. This study also was observed to be the major cause of heterogeneity in the improvement of NIHSS of IVT vs control whether using the event and total or pooled OR. (Supplementary Figs. 5, 6 and 7) This source of heterogeneity is caused due to the difference in population included in this study as it included NIHSS of 0 or 1 only.

Regarding the effect of IVT on NIHSS and the comparison between before and after treatment, Li et al. [8] was observed to be the main source of heterogeneity and this was due to the measurement of NIHSS after 90 days while other studies measured it at discharge so the results weren’t homogenous with the other results. (Supplementary Fig. 8).

Regarding the comparison between IVT and control groups in the change of NIHSS, Logallo et al. [20] was observed to be the main source of heterogeneity among the studies. This was explained by the following: the baseline NIHSS of the control group was significantly lower than that of the intervention group and it remained the same as they received no IVT treatment, so the change was minimal, and it was against the other studies. (Supplementary Fig. 9).

Subgroup analysis was not applicable due to the similarities between the studies in most of the parameters and the homogeneity among the outcomes except for the previously mentioned studies which caused heterogeneity due to the above causes.

## Discussion

This meta-analysis compares the functional outcomes of MIS patients who received IVT with those who did not and evaluates the safety of IVT for MIS by comparing mortality and ICH rates between the two groups. Our findings revealed a significant difference in functional outcomes between MIS patients treated with or without IVT. Patients who received IVT treatment were more likely to have favorable functional outcomes 90 days after MIS. They had higher OR for (mRs 0–1) at 90 days as well as for improvement in NIHSS score when compared with the control group. Additionally, IVT treatment led to a reduction in the NIHSS score for the patients who received it, suggesting an improvement in functional outcomes.

Conflicting evidence exists in the literature regarding the efficacy and safety of IVT in MIS patients. The PRISMS was the first RCT to assess the impact of alteplase treatment in MIS patients. The study found that alteplase did not improve the chances of achieving a favorable functional outcome after 90 days compared with aspirin and was associated with a higher incidence of ICH [13]. This data was in line with a recent meta-analysis comparing IVT and dual antiplatelet therapy [46]. This difference from our study is due to various factors including smaller sample size, different comparison groups such as aspirin in the PRISMS study [13] and dual antiplatelet in the meta-analysis [46] in addition to other factors in the PRISMS study [13] including selection bias and subjective assessment of nondisabling stroke which is based on the assessors and differs between clinicians. Similarly, data from a multicenter nationwide database in China indicated that no significant benefit was observed for alteplase treatment compared with dual antiplatelet therapy or aspirin in MIS patients with a NIHSS score ranging from 0–3 [47].

On the other hand, a separate RCT study known as MINOR suggested that individuals treated with alteplase had notably improved functional outcomes after 90 days compared with those treated with aspirin [48]. A retrospective analysis using data from a multicenter registry database in Korea that involved 1384 minor stroke patients, found that IVT was associated with achieving a favorable outcome at 3 months when compared with those who did not receive IVT treatment [22]. Another retrospective analysis of an inpatient database in the United States, which included 103,765 patients with minor strokes, found that IVT was associated with a higher probability of patients being discharged directly to their homes without requiring assistance [11]. Also, a retrospective study of an Austrian Stroke Unit Registry, which included 890 MIS patients, suggested a potential benefit of IVT in MIS patients [33]. Moreover, Tu et al. [49] in their meta-analysis showed that IVT improves functional outcomes in minor stroke and doesn’t affect mortality when compared with routine medical management. Therefore, the treatment of MIS remains controversial in clinical practice.

Regarding the safety of IVT in MIS, our study showed that there were no statistically significant differences in mortality and ICH rates between the two groups, indicating that IVT is a relatively safe treatment option for MIS patients. These findings are in conformity with the observational studies [11, 22, 33] the MINOR RCT [48] and a previous meta-analysis including seven studies with 1591 patients [50]. There was a fear of the safety and long-term survival of the patients receiving IVT in MIS, however, the current pooled analysis showed that mortality measured at different time points was comparable between IVT and control in addition to comparable risk of the main adverse event of concerns which is ICH. This aligns with previous research which showed that on average, over a 10-year period, a patient receiving thrombolysis survives approximately one year longer than a comparable patient who did not receive thrombolysis, after adjusting for age, sex, prior anticoagulant treatment, acute phase NIHSS score, and subsequent antiplatelet therapy [51]. These findings augment and elaborate on earlier data from the Danish Stroke Register [52] and the subanalysis of the IST-3 trial [53] by illustrating that the advantages of thrombolysis for survival remain significant even up to 10 years post-stroke, while enhanced functional outcomes are still evident at 5 years.

Several factors play a role in the variability among the studies such as: age, gender, territory of the infarct, medical history and chronic medical conditions like hypertension, hyperlipidemia and diabetes. Additionally, the baseline NIHSS score was significantly higher for the IVT group compared with controls in several studies [10, 34, 36, 38]. These factors can immensely affect the functional outcomes in MIS patients. Kim et al. showed that patients with diabetes and deep middle cerebral artery (MCA) territory infarction had a poor prognosis after IVT [54] Moreover, impairment of the motor items in NIHSS was associated with poor outcome in MIS patients [55]. It’s also established that deep white matter or basal ganglia infarction have bad outcomes even after IVT or endovascular therapy (EVT) [56–58]. treatment-related factors, such as differences in the onset-to-IVT time and dosage, may also affect outcomes. Not all the included studies specified the IVT dosage used. Some studies used a dosage of 0.9 mg/kg with a maximum dose of 90 mg, where 10% of the dose is given as an initial bolus and the remaining over one-hour intravenous infusion [35, 36]. Whereas Li et al. [8] used a dosage of 0.6 mg/kg with a maximum dose of 50 mg. The NOR-TEST research conducted in 2017 demonstrated that tenecteplase at a dosage of 0.4 mg/kg is as safe and efficacious as alteplase [59]. Due to the prevalence of mimics or mild strokes among the majority of patients in this study, identifying a significant difference proved challenging. NOR-TEST 2 was conducted to encompass patients with mild-to-severe ischemic stroke [60], revealing that 0.4 mg/kg tenecteplase was associated with elevated rates of sICH, mortality, and disability relative to 0.9 mg/kg alteplase, indicating that 0.4 mg/kg tenecteplase resulted in poorer functional outcomes compared with alteplase. This study determined that tenecteplase at 0.4 mg/kg is less effective than alteplase in treating moderate and severe ischemic strokes. Thus possibly, 0.25 mg/kg tenecteplase might be an alternative dosage among patients with ischemic stroke. The EXTEND-IA TNK trial conducted a direct comparison of 0.4 mg/kg and 0.25 mg/kg dosages of tenecteplase to ascertain the best dosage for individuals experiencing ischemic stroke [61]. They discovered no advantages to 0.4 mg/kg tenecteplase compared with 0.25 mg/kg in ischemic stroke patients. A recent study with 1,600 participants [62] indicated that tenecteplase at a dosage of 0.25 mg/kg is comparable to alteplase at 0.9 mg/kg for functional results, quality of life, and safety for patients with acute ischemic stroke. A recent meta-analysis of nonrandomized data comparing tenecteplase with alteplase found that neither individual RCTs nor prior meta-analyses have raised any safety concerns related to cerebral hemorrhage and death associated with tenecteplase in comparison to alteplase [63].

Not all MIS present have the same severity and personal characteristics. Therefore, it’s important to determine the subset of MIS patients that will benefit the most from IVT treatment.

Regarding the timing of IVT use, the current guidelines and recommendations demonstrated that IVT with recombinant alteplase is advised for individuals who have an acute mild debilitating ischemic stroke lasting less than 4.5 h [64, 65]. CT perfusion and perfusion-diffusion magnetic resonance imaging (MRI) can identify potentially viable brain tissue beyond 4.5 h post-stroke, and reperfusion through thrombolysis has demonstrated improved functional outcomes in patients with salvageable brain tissue beyond this timeframe [66–68]. Regarding the type of stroke, IVT is recommended as a mainstay of treatment in ischemic stroke and should be avoid in hemorrhagic stroke due to the increased risk of bleeding which causes ICH [69].

It seems that MIS patients with infarcts in locations that don’t involve deep MCA territory, deep white matter or basal ganglia have better functional outcomes [54, 56–58]. Also, IVT was not associated with better functional outcomes in MIS patients with a NIHSS baseline score of 0–1 [10]. This is due to the increased risk of early neurological deterioration (END), which can reach up to 14% in patients receiving IVT [70]. The etiology behind END in these cases include reperfusion injury, effects of hyperglycemia, or re-embolization [71].

In patients with MIS, it seems that EVT does not significantly improve functional outcomes when compared with standard medical treatment [72]. However, it's crucial to consider that the effectiveness of EVT as well as IVT can be influenced by various factors, including the severity of the stroke, the location of the clot, the timing of treatment, and individual patient characteristics. EVT is typically reserved for acute ischemic strokes caused by significant vessel occlusions, and its benefits may vary depending on the clinical context.

Our meta-analysis was limited by the number of studies in the literature as well as the variability of data. Moreover, describing what constitutes a "minor" or “mild” stroke in terms of the type and severity of neurological deficits can be challenging due to the lack of a universally agreed-upon definition. To address this issue, some studies use the NIHSS score as a cut-off value to define a minor stroke. However, not all neurological deficits that arise from a minor stroke are effectively assessed using the NIHSS. The scale may not accurately capture strokes in the posterior circulation or the right hemisphere. On the NIHSS, a patient with severe language impairment can have the same score as patient with mild facial weakness, mild dysarthria, and a mild drift of an upper extremity (NIHSS score = 3). However, it is widely agreed among clinicians that a severe language impairment is more disabling for the patient. Therefore, there is a need to redefine what constitutes a minor stroke and how it is diagnosed in clinical practice. Also, the meta-analysis is limited by the predominance of observational studies that act as a major source of bias and no causal relationship, however, limited RCTs regarding this topic is frequently published. This limitation may decrease the evidence of the conclusion, but it reflects real world data of the use of IVT in MIS. Although, the meta-analysis showed homogenous findings in most of the outcomes, some outcomes were observed to have significant heterogeneity. However, this heterogeneity was caused by a single study as we clarified in the sensitivity analysis by leave-one-out methods and provided the reasons for some extreme results.

## Conclusion

While some studies have not found any advantages of IVT in MIS patients, a significant body of literature strongly supports IVT as an effective and safe treatment for MIS. IVT has been shown to improve the mRs and NIHSS scores at the 90-day mark without an increased risk of ICH or mortality. IVT, according to the current analysis, is safe and effective in patients with minor or mild stroke with NIHSS ≤ 5 and this is supported by real world data and large sample size from various centers which can be applied generally. However, there is a need to refine MIS treatment protocols and conduct more RCTs to confirm these findings as RCTs are associated with higher evidence. The current gap in the current research is the most effective and safest dose of IVT drugs to be used in the treatment of MIS and the best timing for it. Also, the risk of END needs further investigation and providing an adequate definition of NIHSS needs to be adjusted across all centers.

## Supplementary Information

Supplementary Material 1. Supplementary Fig. 1: Risk of bias assessment of the included randomized controlled trial using RoB 2.0 tool. Supplementary Fig. 2: Prediction of 90-day mRs 0–1 after using intravenous thrombolysis vs control. Supplementary Fig. 3: Prediction of NIHSS improvement after using intravenous thrombolysis vs control. Supplementary Fig. 4: Comparison between intravenous thrombolysis and control regarding the change in NIHSS. Supplementary Fig. 5: Leave-one-out for the prediction of 90-day mRs 0–1 in intravenous thrombolysis vs control using odds ratio. Supplementary Fig. 6: Leave-one-out analysis for the improvement of NIHSS in intravenous thrombolysis vs control. Supplementary Fig. 7: Leave-one-out for the prediction of NIHSS improvement in intravenous thrombolysis vs control using odds ratio. Supplementary Fig. 8: Leave-one-out for the effect of NIHSS after and before treatment with intravenous thrombolysis. Supplementary Fig. 9: Leave-one-out analysis for the comparison between intravenous thrombolysis and control groups regrading the change in NIHSS.

## Data Availability

All data generated during this systematic review and meta-analysis are included in this published manuscript.

## Abbreviations

MIS: Minor ischemic stroke
IVT: Intravenous thrombolysis
NIHSS: National Institutes of Health Stroke Scale
mRs: Modified Rankin score
r-tPA: Recombinant human tissue-type plasminogen activator
OR: Odds ratio
MD: Mean difference
ICH: Intracerebral hemorrhage
sICH: Symptomatic intracerebral hemorrhage
RCTs: Randomized controlled trials
EVT: Endovascular therapy
CI: Confidence interval
