# Supplementary material for: Safety and efficacy of intravenous thrombolysis: a systematic review and meta-analysis of 93,057 minor stroke patients
Source: BMC Neurol. 2025 Jan 22;25:33. doi: 10.1186/s12883-024-04000-8 (PMC11752810; doi:10.1186/s12883-024-04000-8)
Supplement: Supplementary file 1 — Supplementary Material 1. Supplementary Fig. 1: Risk of bias assessment of the included randomized controlled trial using RoB 2.0 tool. Supplementary Fig. 2: Prediction of 90-day mRs 0–1 after using intravenous thrombolysis vs control. Supplementary Fig. 3: Prediction of NIHSS improvement after using intravenous thrombolysis vs control. Supplementary Fig. 4: Comparison between intravenous thrombolysis and control regarding the change in NIHSS. Supplementary Fig. 5: Leave-one-out for the prediction of 90-day mRs 0–1 in intravenous thrombolysis vs control using odds ratio. Supplementary Fig. 6: Leave-one-out analysis for the improvement of NIHSS in intravenous thrombolysis vs control. Supplementary Fig. 7: Leave-one-out for the prediction of NIHSS improvement in intravenous thrombolysis vs control using odds ratio. Supplementary Fig. 8: Leave-one-out for the effect of NIHSS after and before treatment with intravenous thrombolysis. Supplementary Fig. 9: Leave-one-out analysis for the comparison between intravenous thrombolysis and control groups regrading the change in NIHSS. [file 12883_2024_4000_MOESM1_ESM.docx]

Supplementary table 1: Search strategy applied in the study

| Search strategy | ((Thrombolytic therapy) OR (IVT) OR (rtpa) OR (recombinant tissue plasminogen activator) OR (Actilysis) OR (Fibrinolytic Therapy) OR (Fibrinolysis) OR (intravenous thrombolysis) OR (tissue plasminogen activator) OR (Activase) OR (Clot Buster) OR (Streptokinase) OR (tPA) OR (PLAT) OR (Retaplase) OR (**Alteplase) OR (Tenecteplase) OR (Urokinase) OR (Anistreplase) OR (**urinary-type plasminogen activator) OR (uPA) OR (**Anistreplase) OR (fibrinolysin) OR (IV thrombolysis)) AND ((minor) AND ((stroke) OR (ischemic stroke) OR (**Cerebrovascular Accident) OR (Cerebrovascular Apoplexy) OR (Brain Vascular Accident) OR (CVA) OR (CVAs) OR (Cerebrovascular Stroke) OR (brain attack) OR (cerebral infarction) OR (brain infarction))) |
| --- | --- |

Supplementary table 2: Quality assessment of the included cohort studies using New Castle Ottawa Scale

| **Study ID** | **Selection** | | | | **Comparability** | **Exposure** | | | **Score** | Quality |
| --- | --- | --- | --- | --- | --- | --- | --- | --- | --- | --- |
|  | Is the case definition adequate? | Representativeness of the cases | Selection of Controls | Definition of Controls | Comparability of cases and controls on the basis of the design or analysis | Ascertainment of exposure | Same method of ascertainment for cases and controls | Non-Response rate |  |  |
| Zhong 2021 | * | * | _ | * | * | * | * | * | 7 | High |
| Huisa 2011 | _ | * | _ | _ | ** | * | * | * | 6 | Moderate |
| Hsia 2021 | * | * | * | _ | * | * | * | * | 7 | High |
| Laurencin 2015 | * | * | _ | _ | ** | * | * | _ | 6 | Moderate |
| Li 2019 | * | * | _ | _ | _ | * | * | * | 5 | Moderate |
| Sykora 2021 | * | * | _ | _ | ** | * | * | * | 7 | High |
| Duan 2023 | * | * | * | * | * | * | * | * | 8 | High |
| Villringer 2014 | _ | * | _ | _ | _ | * | * | * | 4 | Moderate |
| Mengel 2022 | * | * | _ | _ | ** | * | * | _ | 6 | Moderate |
| Sharma 2018 | * | * | _ | _ | ** | * | * | _ | 6 | Moderate |
| Han 2021 | * | * | _ | _ | _ | * | * | _ | 4 | Moderate |
| Choi 2015 | * | * | _ | _ | ** | * | * | * | 7 | High |
| Ng 2016 | * | * | _ | _ | * | * | * | _ | 5 | Moderate |
| Greisenegger 2014 | * | * | _ | _ | ** | * | * | _ | 6 | Moderate |
| Logallo 2014 | * | * | _ | _ | ** | * | * | _ | 6 | Moderate |
| Urra 2013 | * | * | _ | _ | * | * | * | _ | 5 | Moderate |
| Chen 2017 | * | * | _ | _ | ** | * | * | _ | 6 | Moderate |
| Luo 2024 | * | * | _ | _ | ** | * | * | _ | 6 | Moderate |
| Yaghi 2018 | * | * | * | _ | * | * | * | _ | 6 | Moderate |
| Pitaksuteepong 2020 | * | * | * | _ | * | * | * | * | 7 | High |


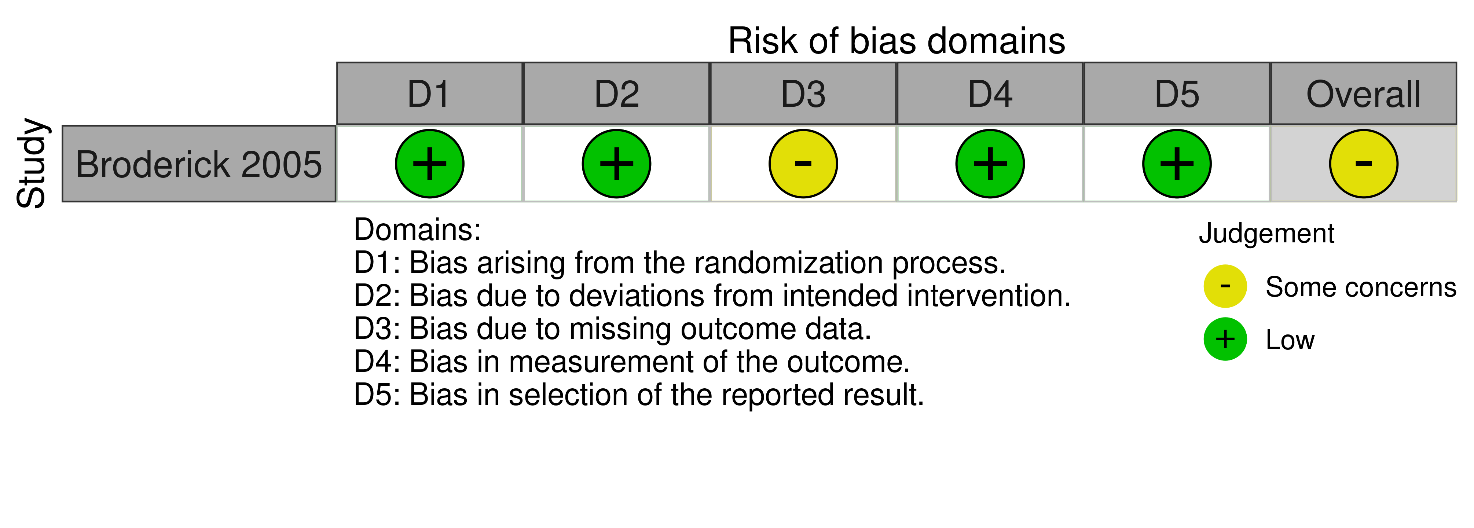


Supplementary figure 1: Risk of bias assessment of the included randomized controlled trial using RoB 2.0 tool


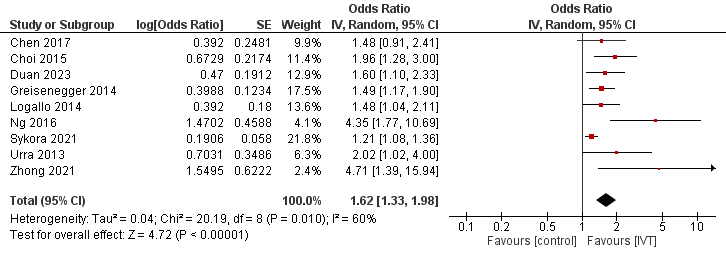


Supplementary figure 2: Prediction of 90-day mRs 0-1 after using intravenous thrombolysis vs control

The diamond shape represents the overall forest plot, the blue region is the OR of each study and the two lines merging from it represent the confidence intervals


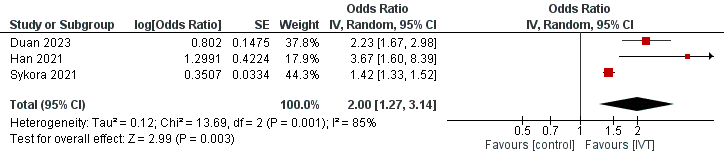


Supplementary figure 3: Prediction of NIHSS improvement after using intravenous thrombolysis vs control

The diamond shape represents the overall forest plot, the blue region is the OR of each study and the two lines merging from it represent the confidence intervals


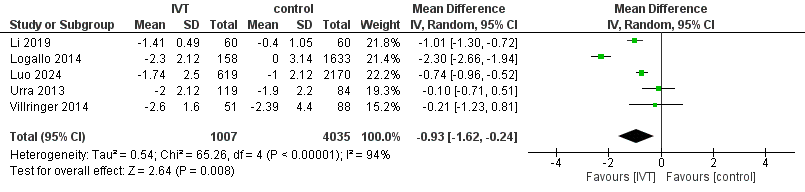


Supplementary figure 4: Comparison between intravenous thrombolysis and control regarding the change in NIHSS

The diamond shape represents the overall forest plot, the blue region is the MD of each study and the two lines merging from it represent the confidence intervals


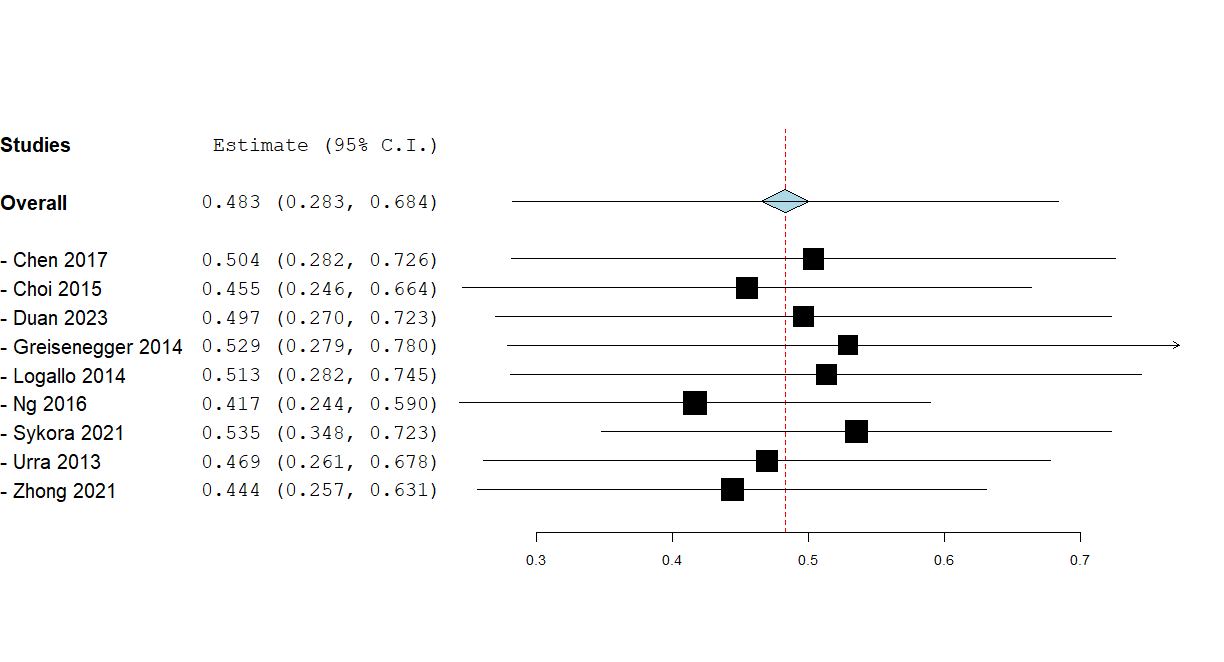


Supplementary figure 5: Leave-one-out for the prediction of 90-day mRs 0-1 in intravenous thrombolysis vs control using odds ratio

The diamond shape represents the overall forest plot, the black region is the OR of each study and the two lines merging from it represent the confidence intervals


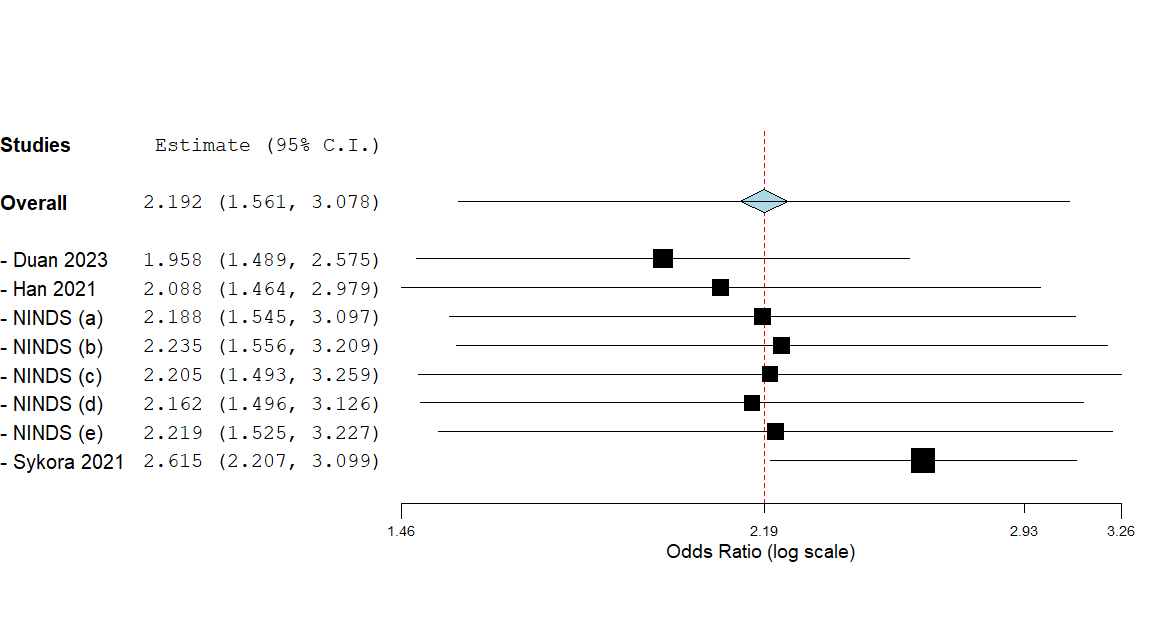


Supplementary figure 6: Leave-one-out analysis for the improvement of NIHSS in intravenous thrombolysis vs control

The diamond shape represents the overall forest plot, the black region is the OR of each study and the two lines merging from it represent the confidence intervals


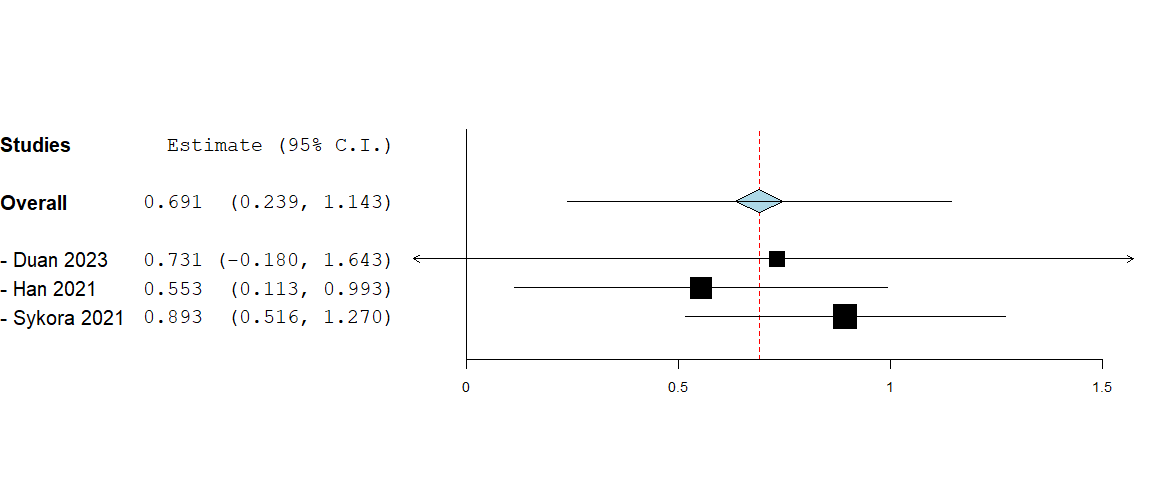


Supplementary figure 7: Leave-one-out for the prediction of NIHSS improvement in intravenous thrombolysis vs control using odds ratio

The diamond shape represents the overall forest plot, the black region is the OR of each study and the two lines merging from it represent the confidence intervals


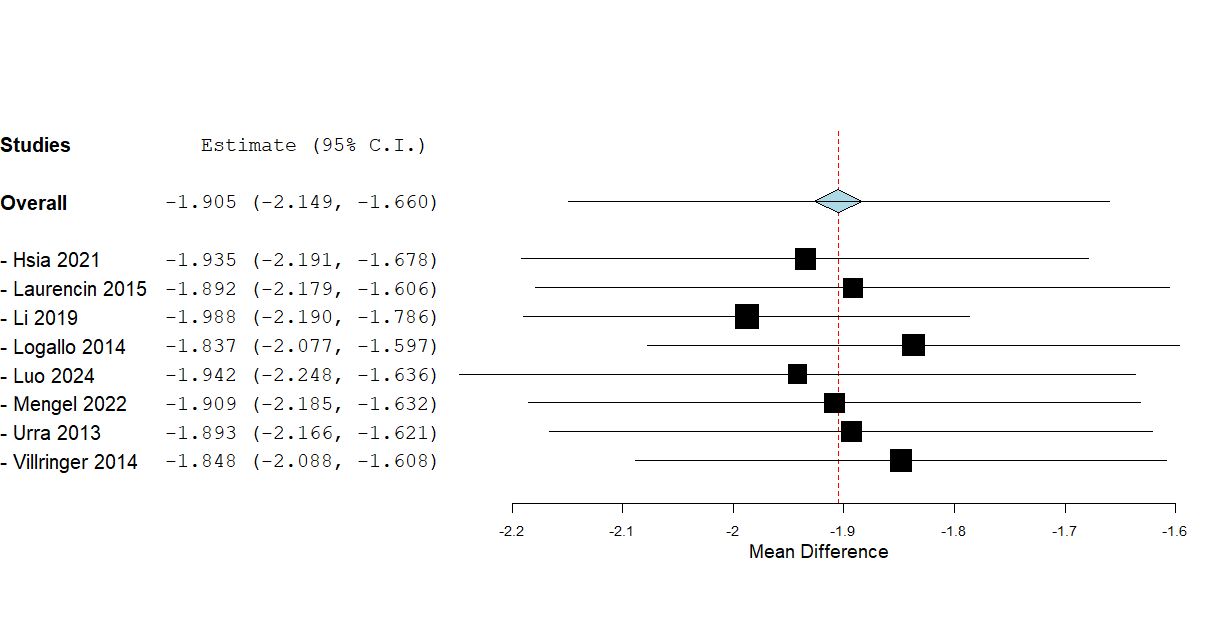


Supplementary figure 8: Leave-one-out for the effect of NIHSS after and before treatment with intravenous thrombolysis

The diamond shape represents the overall forest plot, the black region is the MD of each study and the two lines merging from it represent the confidence intervals


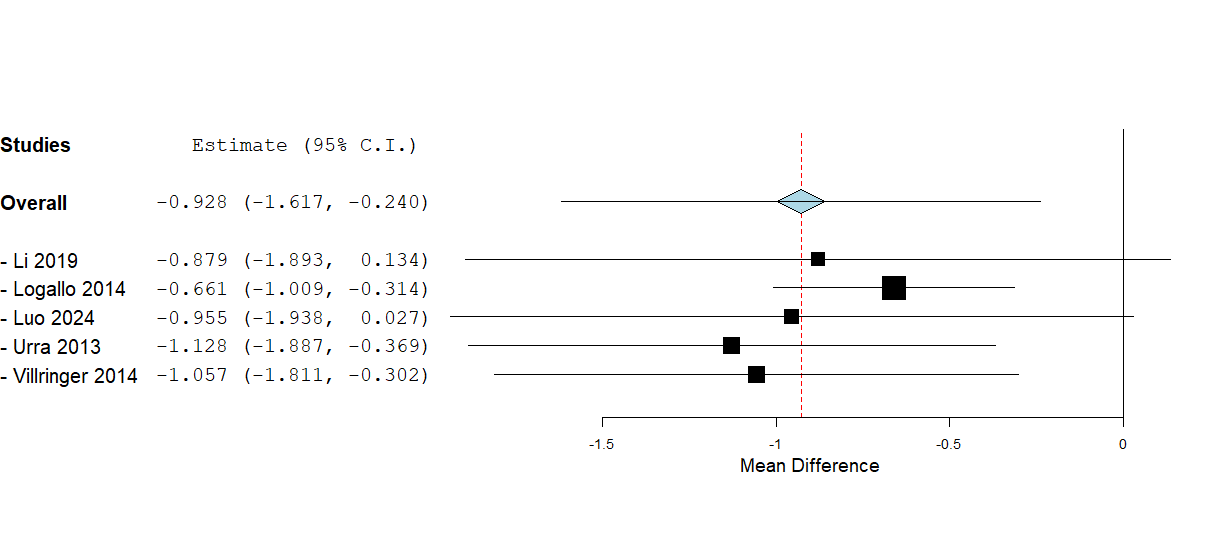


Supplementary figure 9: Leave-one-out analysis for the comparison between intravenous thrombolysis and control groups regarding the change in NIHSS

The diamond shape represents the overall forest plot, the black region is the MD of each study and the two lines merging from it represent the confidence intervals
